# Particulate Air Pollution, Disease, and Death in the Cities and Towns of Southwestern Pennsylvania

**DOI:** 10.5334/aogh.5145

**Published:** 2026-01-28

**Authors:** Ella M. Whitman, Luke Bryan, Sancia Sehdev, Philip J. Landrigan

**Affiliations:** 1 Global Observatory on Planetary Health, Schiller Institute for Integrated Science and Society, Boston College, Chestnut Hill, MA 02467, USA; 2 University of Vermont Larner College of Medicine, Burlington, VT, USA; 3 Centre Scientifique de Monaco, Monaco, MC

**Keywords:** PM_2.5_ air pollution, Pittsburgh, low birth weight, cardiovascular disease, pulmonary disease, stroke, IQ loss, social disadvantage

## Abstract

*Background:* PM_2.5_ air pollution is a leading cause of disease and death. US air pollutant emissions have declined by 75% since passage of the Clean Air Act in 1970, but the Pittsburgh Metropolitan Statistical Area (MSA) continues to have elevated pollution levels and, in 2025, had the US’s 12^th^ highest PM_2.5_ concentration. Steel mills and coke ovens are major point sources.

*Objective:* To quantify deaths, adverse birth outcomes, and children’s IQ loss in the Pittsburgh MSA attributable to PM_2.5_ air pollution.

*Methods:* Mean annual PM_2.5_ air pollution concentrations were obtained for each census tract in the Pittsburgh MSA from NASA’s satellite-based Socioeconomic Data and Applications Center map layers and linked with vital records obtained from the Pennsylvania Department of Health. Exposure–response functions from peer-reviewed literature and EPA’s BenMAP software were used to quantify deaths, adverse birth outcomes, and IQ loss attributable to PM_2.5_ pollution.

*Results:* The mean annual PM_2.5_ concentration in the Pittsburgh MSA was 8.54 μg/m^3^. Concentrations across census tracts ranged from 5.74 to 15.90 μg/m^3^. Of 27,224 adult deaths in the Pittsburgh MSA in 2019, we estimate that between 3,085 and 3,467 (11.1%–12.5%) were attributable to PM_2.5_ pollution. We estimate that 229 premature births, 177 low-weight births, and 12 stillbirths could be attributed to prenatal PM_2.5_ exposure. Among the 24,604 children born in the Pittsburgh MSA in 2019, PM_2.5_ pollution was linked to the loss of 60,668 full-scale IQ points, resulting in estimated lifetime economic losses of $2.7 billion.

*Conclusion:* In 2019, 11.1%–12.5% of adult deaths in the Pittsburgh MSA, more than 400 adverse birth outcomes, and widespread reductions in children’s IQ were attributable to PM_2.5_ air pollution. Public policies and strict enforcement that reduce pollutant emissions and improve air quality will improve the health of southwestern Pennsylvania residents, save lives, and be highly cost-effective.

## Introduction

Fine particulate matter (PM_2.5_) air pollution is a major cause of disease, disability, and premature death. It is responsible for an estimated 197,000 deaths (95% CI, 183,000–214,000) each year in the United States [1] and for at least 7 million deaths globally [2]. Fossil fuel combustion is the predominant source of PM_2.5_ pollution and accounts for 85% of airborne particulate emissions [3].

PM_2.5_ pollution harms human health from infancy to old age and is linked to multiple non-communicable diseases [4]. In adults, these include cardiovascular disease, stroke, chronic obstructive pulmonary disease, lung cancer, and diabetes [4–11]. In infants and children, air pollution increases risk for premature birth [12–14], low birthweight [12–14], stillbirth [12–14], asthma [15–17], and impaired lung development [18].

PM_2.5_ pollution is additionally associated with neurologic dysfunction. In adults, it is reported to increase the risk of dementia [19–20], while in children it is linked to loss of cognitive function (IQ loss), memory deficits, behavioral dysfunction, reductions in brain volume, and increased risks of attention deficit/hyperactivity disorder (ADHD) and autism spectrum disorder (ASD) [21–33]. IQ loss is associated with reduced educational attainment, diminished economic productivity, and decreased lifetime earnings [33].

Many of the adverse health effects attributed to PM_2.5_ pollution have been documented at levels of exposure below the US Environmental Protection Agency’s current annual air quality standard (NAAQS) of 9.0 μg/m^3^ air, expressed as an annual mean [10, 34], and below the World Health Organization’s recommended guideline of 5 μg/m^3^ air [4]. Emerging data indicate that the relationship between PM_2.5_ pollution and disease extends down to the lowest measurable levels of exposure.

Southwestern Pennsylvania and Pittsburgh, its largest city, have long been centers of heavy industry, particularly coal mining, steel making, and more recently hydraulic fracturing (fracking) for oil and gas [35]. In consequence, the region has a history of significant air and soil pollution that is exacerbated by its distinct topographical and meteorological features, especially its steep valleys and frequent winter inversions [36–41]. The worst recorded acute air pollution disaster in the United States occurred in Donora PA, just outside Pittsburgh in 1948 [36]. It resulted in multiple deaths and hospitalizations and was an important catalyst for both air pollution research, which continues in Pittsburgh to the present day [40, 41], and of clean air legislation.

While air pollutant emissions have decreased by 75% across the United States since the passage of the Clean Air Act in 1970 [42], the Pittsburgh Metropolitan Statistical Area (MSA) continues to experience elevated concentrations of PM_2.5_ and other air pollutants, such as black carbon and hazardous chemical pollutants. In 2025, the Pittsburgh MSA had the 12^th^-worst air quality in the United States [39]. Industrial point sources, notably coke works and steel mills, along with motor vehicles, are the main regional sources of air pollution.

This report presents an epidemiologic study undertaken by Global Observatory on Planetary Health at Boston College quantifying the burden of disease, death, and pediatric IQ loss associated with PM_2.5_ air pollution in the cities and towns of the Pittsburgh MSA. The study also examines associations between PM_2.5_ air pollution, social disadvantage, and health impairment. It presents recommendations for air quality improvement to improve health.

## Methods

### Overview

We used a geographically fine-grained, cross-sectional study design to quantify the burden of mortality, adverse birth outcomes, and pediatric IQ loss attributable to PM_2.5_ air pollution within the eight counties of the Pittsburgh MSA in calendar year 2019. We linked estimates of annual mean PM_2.5_ concentrations in each census tract with geocoded individual vital records, applying exposure–response functions derived from the peer-reviewed epidemiologic literature in EPA’s BenMAP software platform. We examined associations between PM_2.5_ air pollution, social disadvantage, and health impairment. Our detailed methodology is presented in the following paragraphs.

This study was approved by the Boston College Institutional Review Board (IRB) (protocol 24.085.01e-1) and the Pennsylvania Department of Health IRB (protocol 1C-3078).

### Population estimates

We utilized data published by the US Census Bureau to estimate the population in each census tract within the Pittsburgh MSA. This MSA encompasses eight counties in southwestern Pennsylvania: Allegheny, Armstrong, Beaver, Butler, Fayette, Lawrence, Washington, and Westmoreland.

### PM_2.5_ exposure estimates

Mean annual airborne PM_2.5_ concentrations for each census tract within the Pittsburgh MSA were obtained from NASA’s satellite-based Socioeconomic Data and Applications Center (SEDAC) map layers [43]. We used PM_2.5_ concentration data from 2016, the most recent year for which data were available. These data provided PM_2.5_ estimates at a highly localized grid resolution of 1 x 1 km [44]. We calculated annual mean PM_2.5_ concentrations at the census-tract level by averaging all point estimates within each census tract. Of the 739 census tracts investigated, 41 contained no point estimates, and we therefore used the nearest PM_2.5_ estimate for those tracts’ annual mean concentrations. This analysis assumed planar geography using an NAD83 projection, the most appropriate for the region. All analyses were conducted using R Statistical Software (v4.4.1; R Core Team 2024).

### Health outcomes

To assess PM_2.5_ pollution’s impacts on morbidity and mortality, we obtained vital statistics data, including death certificates, birth certificates, and fetal death files from the Pennsylvania Department of Health, Bureau of Health Statistics and Registries for all decedents, live births, and stillbirths in the Pittsburgh MSA for 2019. Death certificates included information on each individual’s residential address and underlying cause of death (specified by ICD-10) [45]. Birth files contained residential address at the time of birth, length of pregnancy, and birth weight. Fetal death files recorded the birthing parent’s residential address. All residential addresses were geocoded.

We selected health endpoints *a priori* for inclusion in this analysis based on published epidemiologic studies in the peer-reviewed literature that credibly linked adverse health effects with PM_2.5_ air pollution [9, 11, 13, 14, 46–48]. The adult health outcomes included were all-cause mortality and cause-specific mortality from lung cancer, ischemic heart disease (IHD), and myocardial infarction (a subset of IHD). The adverse birth outcomes included were preterm birth (<37 weeks’ gestation), low birth weight (< 2,500 g), and stillbirth. Additionally, we quantified IQ loss among children in the 2019 birth cohort [33] and its associated lifetime economic impacts [49].

To quantify the burden of disease and death associated with PM_2.5_ pollution exposure, we used concentration–response functions (*β* coefficients) from the published epidemiologic literature [9, 11, 13, 14, 46–48] (see Supplementary Appendix). In the case of endpoints for which there were multiple high-quality studies with varying *β* coefficients, we used more than one concentration–response function and present a range of estimates.

When effect estimates were reported as odds ratios (OR) or relative risks (RR) for a specified PM_2.5_ increment, we converted these estimates into *β* coefficients (concentration–response functions). This conversion was performed by taking the natural logarithm of the reported OR or RR and dividing it by the PM_2.5_ increment used in the original study. Each *β* coefficient then represents the change in the frequency of an outcome observed per 1 μg/m³ increase in the annual mean PM_2.5_ concentration.

By applying these *β* coefficients to our PM_2.5_ exposure estimates and demographic data, we were able to calculate the number and the proportion of each health outcome attributable to PM_2.5_ air pollution in each census tract in the Pittsburgh MSA, thus providing a geographically fine-grained quantitative estimate of the burden of air pollution-related disease, disability, and premature mortality.

### IQ loss

To assess neurocognitive impairment (IQ loss) among children in the Pittsburgh MSA due to prenatal and early childhood exposure to PM_2.5_ air pollution, we used data from a systematic review and meta-analysis conducted by Alter and Whitman [33]. This study found that each 1 μg/m^3^ increase in PM_2.5_ concentration is associated with a 0.27-point loss in full-scale IQ (FSIQ), a 0.24-point loss in verbal IQ (VIQ), and a 0.40-point loss in performance IQ (PIQ). VIQ reflects a child’s communication and language skills, whereas PIQ reflects non-verbal cognitive abilities such as the ability to reason and to solve novel problems [33]. Full-scale IQ is a combined metric, based on characteristics captured in the PIQ and VIQ assessments.

### Economic impact of PM_2.5_-associated IQ loss

We estimated the total lifetime economic impact of PM_2.5_-attributable FSIQ loss among children in the Pittsburgh MSA 2019 birth cohort by applying the monetary value of one IQ point taken from Grosse and Zhou [49]. These investigators estimate that the present value of lifetime earnings per IQ point is $10,600–13,100 in the United States. In our analysis, we used this range of values to estimate the mean and the lower and upper limits of economic loss attributable to the loss of FSIQ points.

### Social disadvantage estimates

We estimated the social disadvantage of each census tract in the Pittsburgh MSA using the Area Deprivation Index (ADI), developed by Kind and colleagues at the University of Wisconsin [50]. The ADI is a composite measure based on 17 indicators of socioeconomic status across 4 domains—employment, income, education, and housing quality—that enables neighborhood ranking by socioeconomic disadvantage. In this analysis, we stratified the 2008 census block groups in the Pittsburgh MSA into deciles using data from the 2021 ADI.

### Statistical analysis

We used the U.S. Environmental Protection Agency’s open-source Environmental Benefits Mapping and Analysis Program (BenMAP-CE) tool [51] to estimate the number of deaths and adverse birth outcomes attributable to PM_2.5_ pollution exposure in each census tract in the Pittsburgh MSA. The Health Impact Function (HIF) in BenMAP-CE uses *β* coefficients obtained from published epidemiological studies to quantify the expected change in a health endpoint per unit increase in PM_2.5_ concentration in a given population, based on the baseline incidence of a disease in the population, the population size and demographics, and the estimated PM_2.5_ exposure level. The HIF is expressed as:


$$\Delta Y=(1-e^{-\beta \times \Delta AQ})\times Yo\times Pop$$

where Δ*Y* represents the number of cases of a particular adverse outcome associated with a change in PM_2.5_ levels. The beta coefficient (*β*) is an exposure–response function obtained from the epidemiologic literature. The baseline incidence rate (*Y*₀) represents the background rate of the health outcome of interest in the population, while Pop denotes the size of the population exposed to PM_2.5_ based on US Census data. Change in air quality (ΔAQ) is defined as the difference between the calculated annual mean PM_2.5_ concentration at the census-tract level and the comparison (counterfactual) concentrations, which we set to 0 μg/m³ in this analysis. These parameters allow for estimation of the number of cases of a particular health outcome attributable to PM_2.5_ exposure within the study area. Additionally, to calculate the number of deaths that could be prevented by lowering the annual mean PM_2.5_ concentration across the region to the World Health Organization’s air pollution guideline of 5 μg/m³, we reran our calculations using that value as the counterfactual (see Supplementary Appendix). BenMAP-CE outputs are generated at the census-tract level, and R statistical software is used to aggregate these results to the county level.

### Map generation

We used the Leaflet package in R to develop maps displaying the burden of disease attributable to PM_2.5_ air pollution in each census tract in the Pittsburgh MSA in 2019. To identify major point sources of pollution emissions in these maps, we relied on data from US EPA’s 2014 National Emission Inventory (NEI) for information on air pollution levels and sources [52]. Three pollution emission sources of particular interest are the Clairton Coke Works, the Irvin Works, and the Edgar Thompson Works, all located in Allegheny County [39–41, 53].

## Results

### Population

The estimated population of the Pittsburgh MSA in 2019 was 2,418,174, with 1,221,744 of these persons residing in Allegheny County and the remainder in the other 7 counties of the region. In 2019, there were 24,604 live births in the Pittsburgh MSA, with 12,834 births in Allegheny County. In the same year, there were 28,510 deaths in the Pittsburgh MSA, with 27,224 of these deaths in adults 25–99 years of age (Table 1).

**Table 1:** Population, birth and death data, and annual mean PM_2.5_ concentrations by County, Pittsburgh MSA, 2019.

COUNTY	POPULATION	LIVE BIRTHS	DEATHS	MEAN PM_2.5_ CONCENTRATION
Allegheny	1,221,744	12,834	13,467	9.77
Armstrong	65,867	2,092	859	8.15
Beaver	165,833	1,906	2,153	8.37
Butler	186,899	1,683	2,085	8.38
Fayette	131,302	3,202	1,730	8.95
Lawrence	86,727	903	1,128	8.26
Washington	207,212	1,339	2,577	7.92
Westmoreland	352,590	645	4,511	8.53
**Pittsburgh MSA**	**2,418,174**	**24,604**	28,510*	**8.54**

*Note*: 27,224 deaths in the Pittsburgh MSA occurred in adults 25–99 years of age. Pollution-attributable fractions of deaths were calculated in this age range.

### PM_2.5_ exposure

The annual mean PM_2.5_ concentration across the eight counties of the Pittsburgh MSA in 2019 was 8.54 ± 0.46 μg/m^3^, ranging from a low of 5.74 to a high of 15.90 μg/m^3^ across all census tracts. Annual mean PM_2.5_ concentrations in many census tracts were below the EPA standard of 9.0 μg/m^3^.

**Figure 1:**
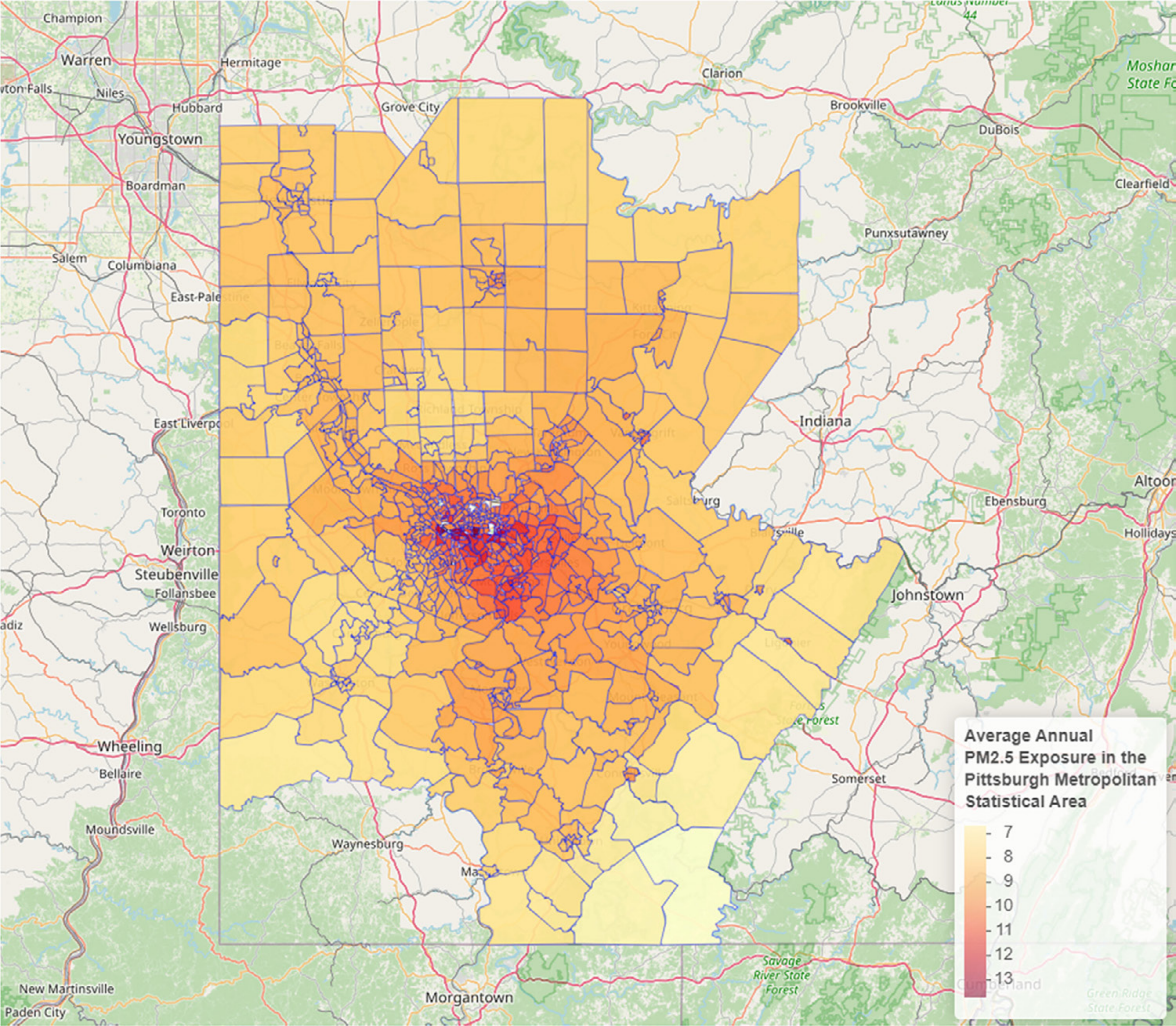
Estimated annual mean PM_2.5_ exposure by census tract, Pittsburgh MSA, 2016.

**Figure 2:**
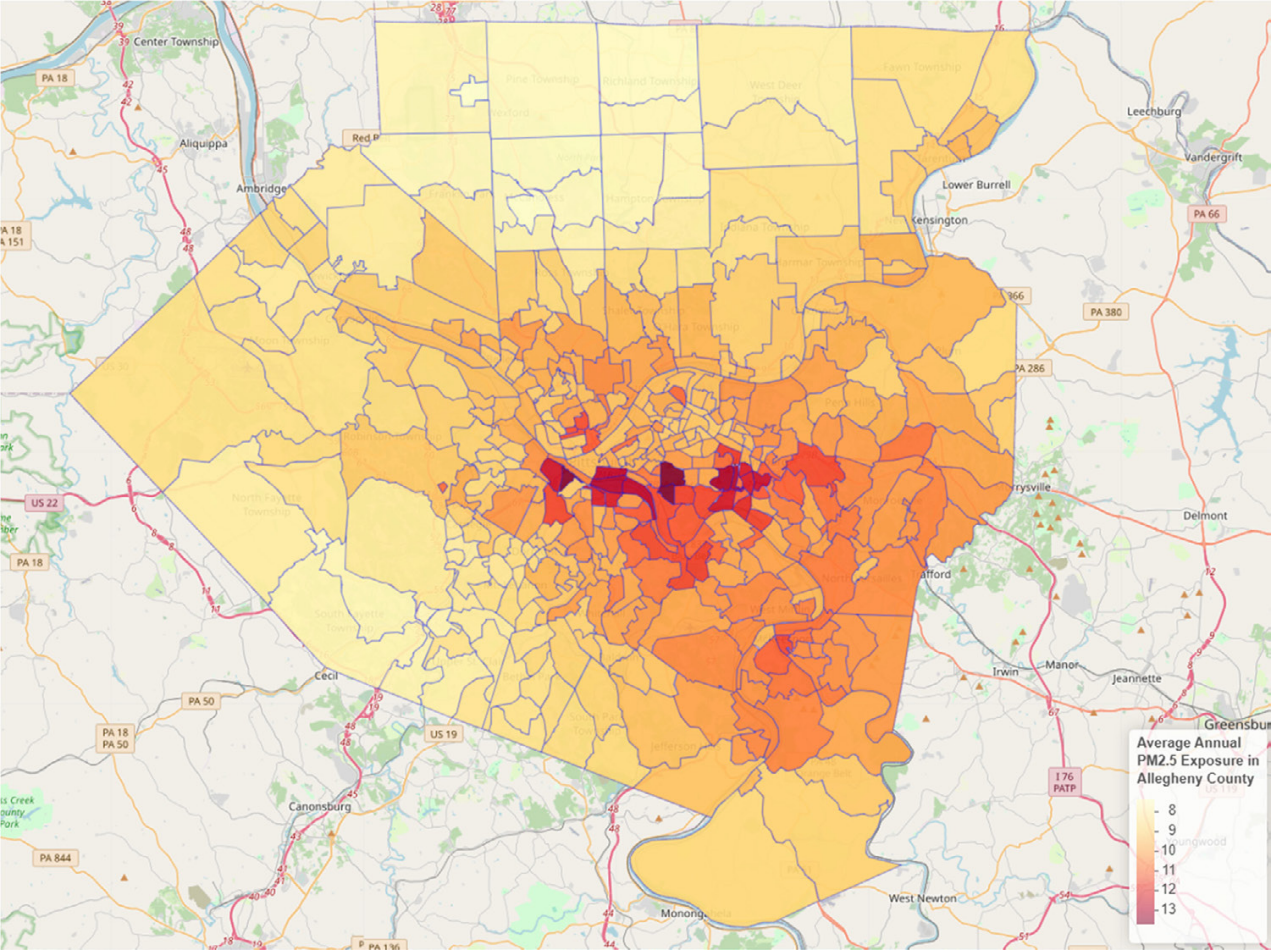
Estimated annual mean PM_2.5_ exposure by census tract, Allegheny County, PA, 2016.

Substantial variation in annual mean PM_2.5_ concentrations was observed within and between counties reflecting the geographical distribution of stationary emission sources such as factories and power plants and major roadways (Figure 1). At the county level, annual mean PM_2.5_ concentrations ranged from a low of 7.92 μg/m^3^ in Washington County to a high of 9.77 μg/m^3^ in Allegheny County (Table 1). Within Allegheny County, highest annual mean PM_2.5_ concentrations were seen south and east of Pittsburgh (Figure 2).

### Health outcomes

*Mortality.* We estimate that between 3,085 and 3,467 (11.1%–12.5%) of the 27,724 adult deaths that occurred in the Pittsburgh MSA in 2019 could be attributed to PM_2.5_ air pollution. These included 396 deaths from lung cancer; 702 deaths from IHD; and 65 deaths from myocardial infarction (a wholly contained subset of IHD deaths) (Table 2).

**Table 2:** PM_2.5_-Attributable mortality, by underlying cause, Pittsburgh MSA, 2019.

	ALL-CAUSE MORTALITY (A00 – Z99)				LUNG CANCER (C34)		ISCHEMIC HEART DISEASE (IHD) (I20 – I25)		MYOCARDIAL INFARCTION (SUBSET OF IHD) (I21)	
	LEPEULE [46]		LADEN [9]		GHARIBVAND [47]		KREWSKI [48]		ALEXEEFF [11]	
COUNTY	COUNTS	PERCENT ATTRIBUTABLE	COUNTS	PERCENT ATTRIBUTABLE	COUNTS	PERCENT ATTRIBUTABLE	COUNTS	PERCENT ATTRIBUTABLE	COUNTS	PERCENT ATTRIBUTABLE
Allegheny	1,553	11.86%	1,744	13.32%	189	29.20%	382	18.72%	32	7.17%
Armstrong	85	10.54%	95	11.85%	12	26.36%	16	16.76%	2	6.44%
Beaver	219	10.41%	246	11.71%	27	25.94%	44	16.52%	5	6.26%
Butler	210	10.41%	236	11.71%	28	25.84%	41	16.56%	4	6.25%
Fayette	166	9.89%	187	11.12%	24	24.65%	40	15.75%	3	5.96%
Lawrence	113	10.26%	127	11.54%	15	25.53%	23	16.22%	2	6.14%
Washington	252	10.09%	283	11.35%	41	25.01%	52	16.35%	6	6.07%
Westmoreland	488	11.00%	548	12.36%	60	27.10%	104	17.38%	11	6.54%
**Pittsburgh MSA**	**3,085**	11.13%	**3,467**	12.51%	**396**	27.37%	**702**	17.72%	**65**	6.69%

**Figure 3:**
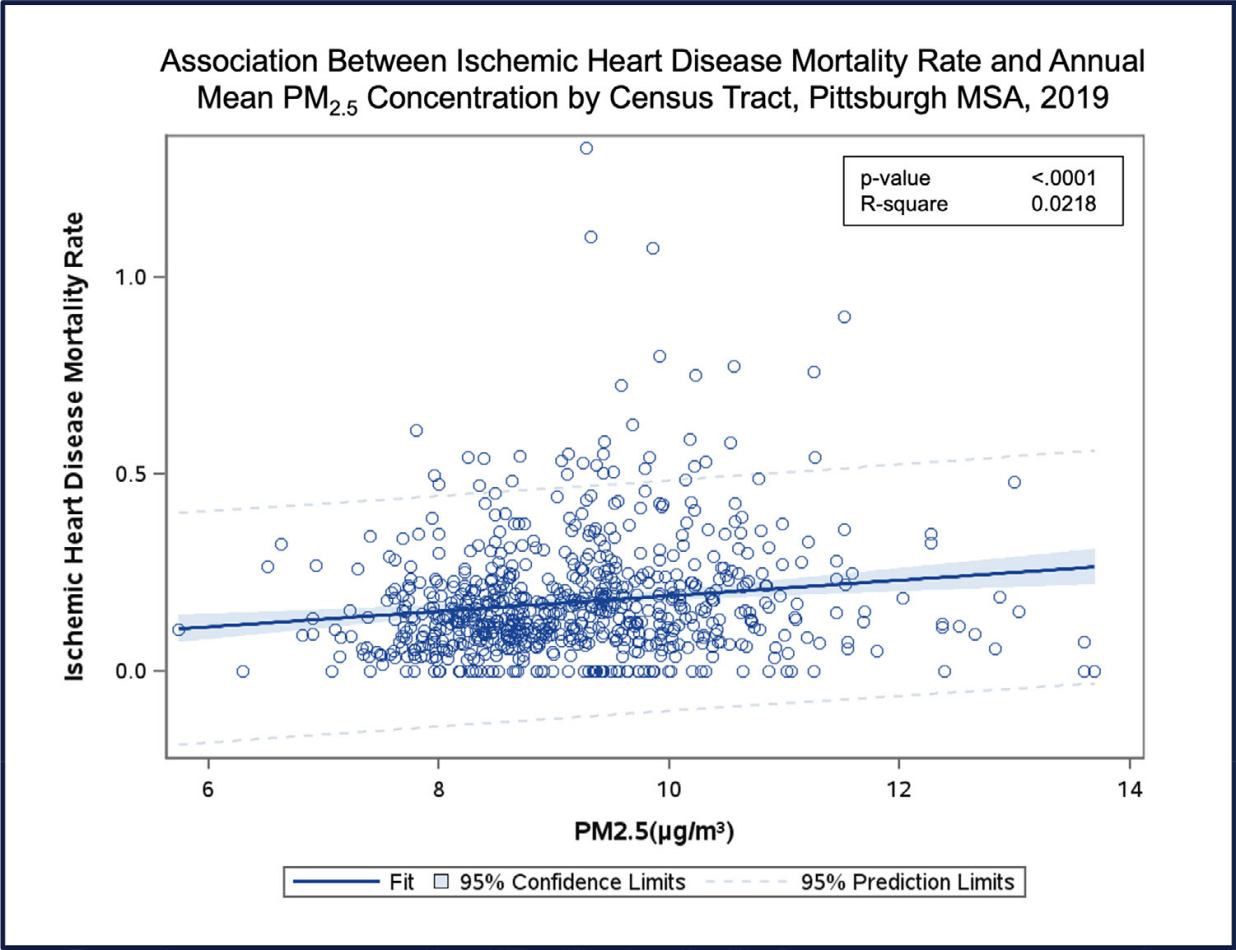
Association between ischemic heart disease mortality rate and annual mean PM_2.5_ concentration by census tract, Pittsburgh MSA, 2019. * Rates were calculated using ischemic heart disease deaths and population counts within the at-risk population of each census tract, using the correlation coefficient from the Krewski et al. study [48].

We observed positive exposure–response relationships between annual mean PM_2.5_ concentrations and major causes of mortality (Figure 3). Mortality rates were calculated using disease-specific deaths and population counts within the at-risk population of each census tract.

**Figure 4:**
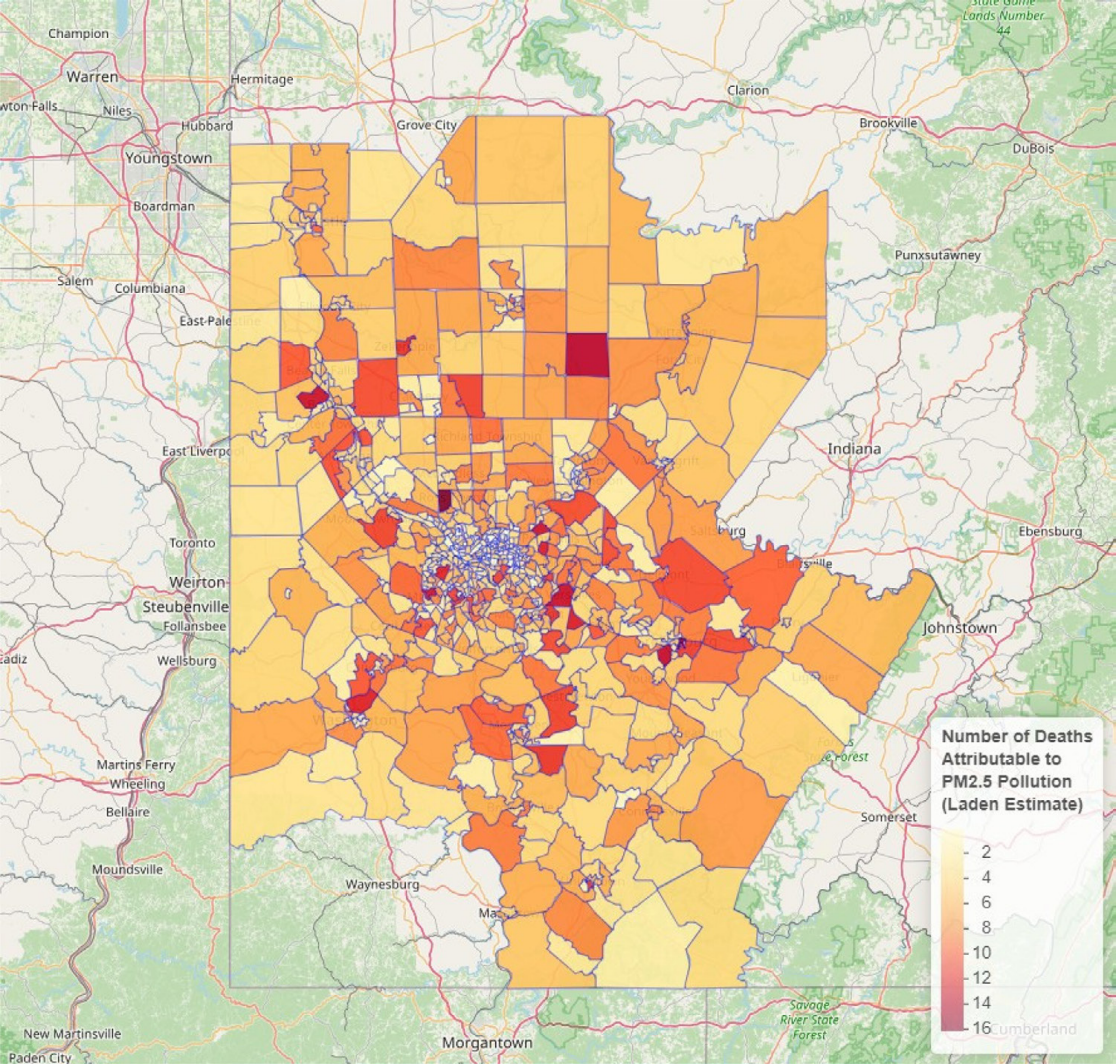
PM_2.5_-attributable all- cause mortality by census tract, Pittsburgh MSA, 2019. Note: Estimates generated using Laden et al. [9].

**Figure 5:**
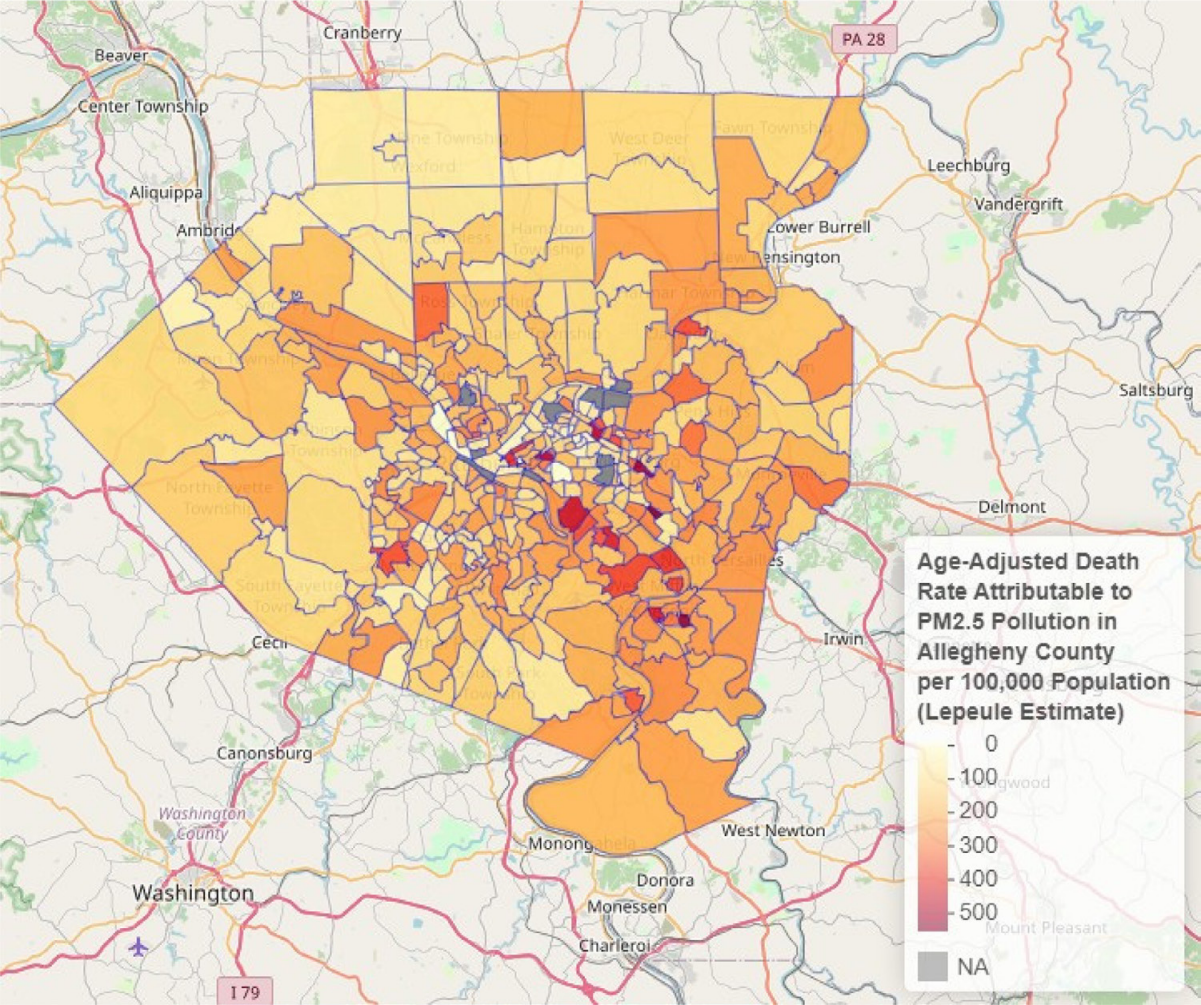
PM_2.5_-attributable all- cause mortality by census tract, Allegheny County, PA, 2019. **Note**: Estimates generated using Lepeule et al. [46].

The largest numbers of PM_2.5_-attributable deaths in each of the above categories occurred in Allegheny County. Within and between counties there was substantial geographic variation, with highest PM_2.5_-attributable death rates seen in the census tracts with highest annual mean PM_2.5_ concentrations (Figure 4). Within Allegheny County, highest annual mean PM_2.5_ concentrations were seen south and east of Pittsburgh (Figure 5).

**Figure 6:**
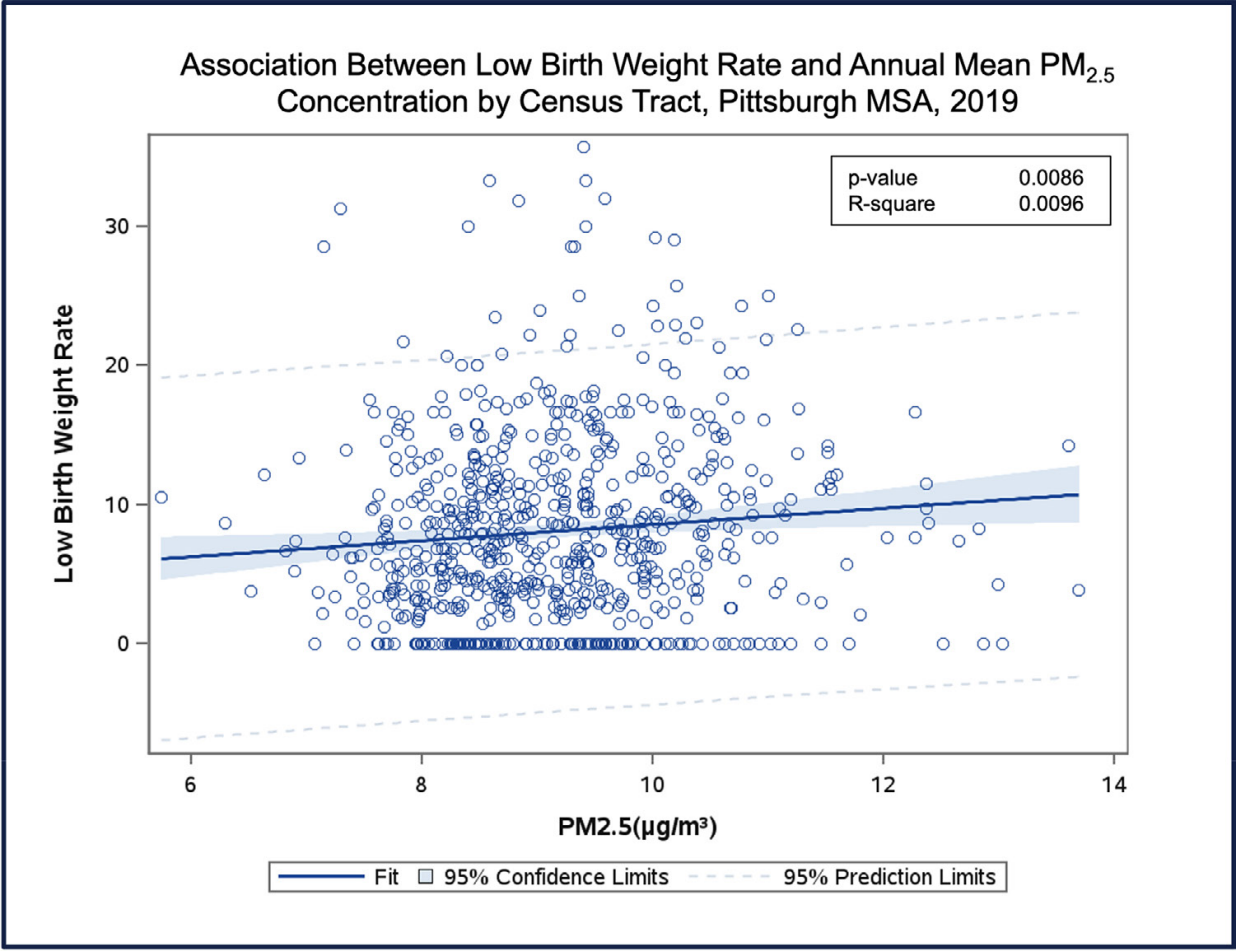
Association between low-birth-weight rate and annual mean PM_2.5_ concentration by census tract, Pittsburgh MSA, 2019.

*Adverse birth outcomes.* We estimate that 229 premature births (<37 weeks’ gestation), 177 low-birth-weight babies (<2,500 g), and 12 stillbirths (fetal deaths) in the Pittsburgh MSA in 2019 could be attributed to PM_2.5_ air pollution (Table 3). We observed positive exposure–response relationships between annual mean PM_ 2.5_ concentrations and adverse birth outcomes (Figure 6).

**Table 3:** PM_2.5_-attributable adverse birth outcomes, Pittsburgh MSA, 2019.

	PRETERM BIRTH GHOSH [13]		LOW BIRTH WEIGHT GHOSH [13]		STILL BIRTHS ZHANG [14]	
COUNTY	COUNTS	PERCENT ATTRIBUTABLE	COUNTS	PERCENT ATTRIBUTABLE	COUNTS	PERCENT ATTRIBUTABLE
Allegheny	129	10.4%	102	9.6%	6	8.3%
Armstrong	17	8.6%	12	8.0%	1	6.9%
Beaver	14	9.0%	10	8.3%	1	7.3%
Butler	13	9.1%	10	8.4%	1	7.3%
Fayette	26	9.6%	20	8.9%	1	7.6%
Lawrence	9	8.9%	6	8.2%	1	7.2%
Washington	14	8.5%	10	7.9%	1	7.3%
Westmoreland	7	9.1%	7	8.4%	0	7.2%
**Pittsburgh MSA**	**229**	**9.7%**	**177**	**9.0%**	**12**	**7.8%**

*Note:* Preterm birth defined as <37 weeks’ gestation and low birth weight as <2,500 g, as per WHO guidelines.

**Figure 7:**
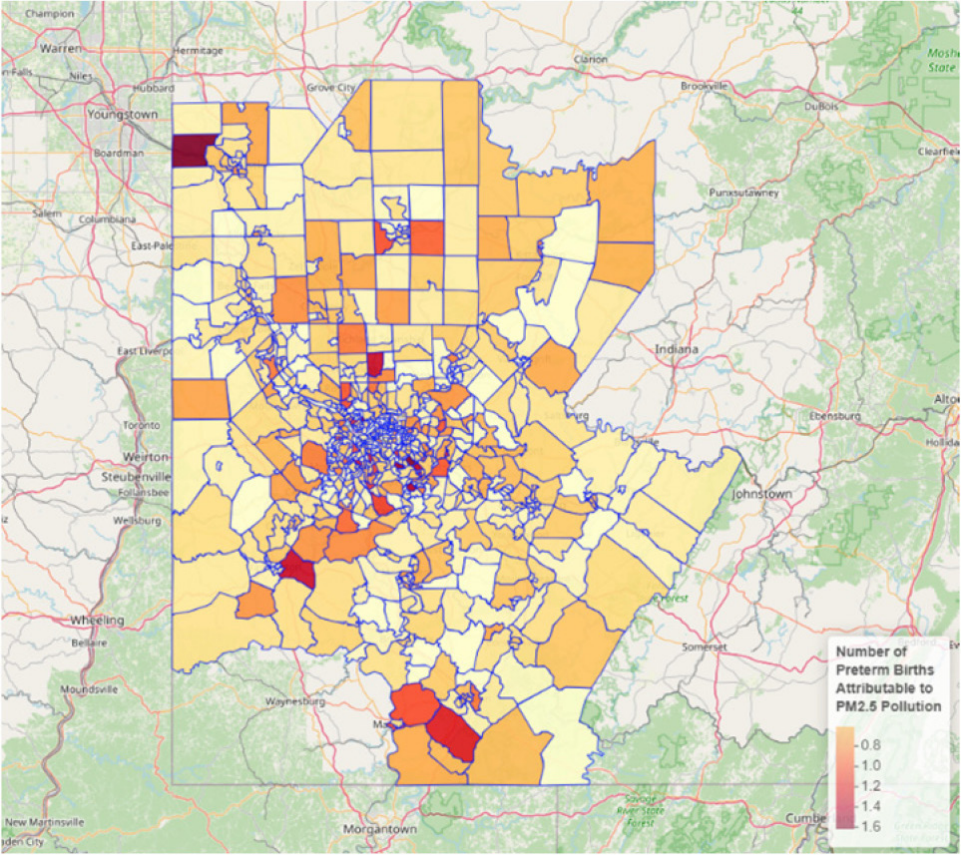

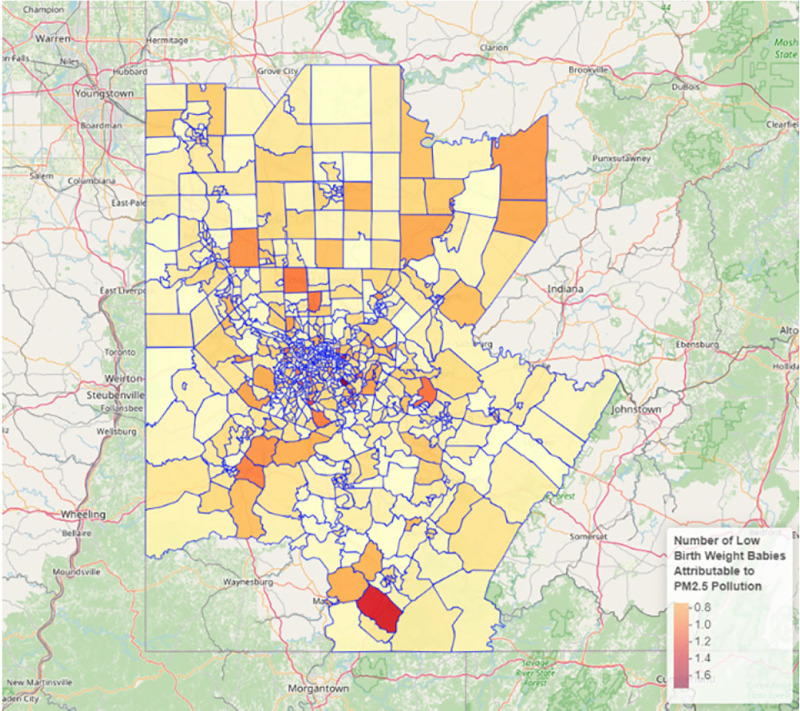

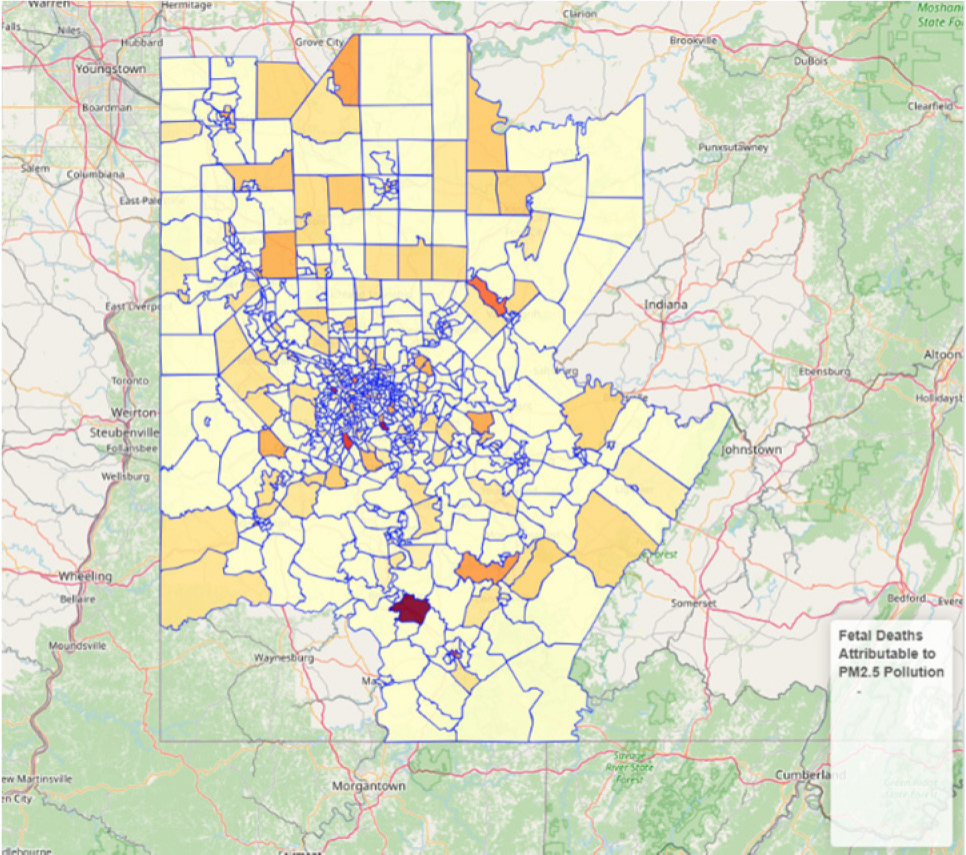
PM_2.5_-attributable adverse birth outcomes by census tract, Pittsburgh MSA, 2019.

The largest numbers of each of these PM_2.5_-attributable adverse birth outcomes occurred in Allegheny County. Within and between counties there was substantial geographic variation, with highest rates of adverse birth outcomes seen in the census tracts with highest mean PM_2.5_ concentrations (Figure 7).

### IQ loss and its economic impact

We estimate that the loss of 60,668 FSIQ points, 53,927 VIQ points, and 87,631 PIQ points in the 24,604 children in the Pittsburgh MSA 2019 birth cohort could be attributed to these children’s early-life exposures to PM_2.5_ air pollution (Table 4).

**Table 4:** PM_2.5_-attributable IQ loss among children, Pittsburgh MSA, 2019 birth cohort.

COUNTY	FSIQ	VIQ	PIQ
Allegheny	33,848	30,087	48,891
Armstrong	4,606	4,094	6,653
Beaver	4,308	3,829	6,223
Butler	3,808	3,385	5,500
Fayette	7,735	6,876	11,173
Lawrence	2,013	1,789	2,907
Washington	2,865	2,547	4,138
Westmoreland	1,485	1,320	2,145
**Pittsburgh MSA**	**60,668**	**53,927**	**87,631**

*Note:* Estimates of IQ points lost per 1 μg/m³ increase in PM_2.5_ were derived from Alter et al. [33] with a counterfactual concentration set to 0 μg/m.

This loss of 60,668 full-scale IQ points will result in reduction of these children’s lifetime earnings by an estimated $2.7 billion (95% CI, 2.3–2.9) across their lifetimes [49] (Table 5).

**Table 5:** Lifetime economic impact of PM_2.5_-attributable full-scale IQ loss among children, Pittsburgh MSA, 2019 birth cohort.

COUNTY	AVERAGE ECONOMIC LOSSES (US$)	LOWER LIMIT ECONOMIC LOSSES (US$)	UPPER LIMIT ECONOMIC LOSSES (US$)
Allegheny	1,485,541,661	1,328,838,954	1,642,244,368
Armstrong	202,155,256	180,830,862	223,479,650
Beaver	189,073,370	169,1289,22	209017819
Butler	167,118,473	149,489,942	184,747,004
Fayette	339,478,376	303,668,421	375,288,331
Lawrence	88,341,376	79,022,665	97,660,087
Washington	125,738,659	112,475,087	139,002,231
Westmoreland	65,185,718	58,309,587	72,061,848
**Pittsburgh MSA**	**2,662,632,888**	**2,381,764,440**	**2,943,501,336**

*Note:* The monetary valuation of one IQ point was obtained from Grosse and Zhou [49], who estimated the present value of lifetime earnings in the United States at US$ 10,600–13,100 per IQ point. In our analysis, US$ 10,600 and US$ 13,100 were used to calculate the lower and upper limits, respectively, and the Average Economic Loss was calculated as their mean. All economic estimates are based on PM_2.5_-attributable FSIQ points lost.

### Social disadvantage

In southwestern Pennsylvania, the 10% of census block groups with highest levels of social deprivation had the highest annual mean PM_2.5_ concentrations—9.58 μg/m³, whereas the least deprived (most affluent) neighborhoods had the lowest annual mean PM_2.5_ concentration—8.70 μg/m³ [54].

## Discussion

The main finding of this analysis is that between 3,085 and 3,467 (11.1%–12.5%) adult deaths in the 8 counties of the Pittsburgh MSA in 2019 as well as 229 premature births, 177 low-weight births, and 12 stillbirths could be attributed to fine particulate matter (PM_2.5_) air pollution. Additionally, the loss of an estimated 60,668 full-scale IQ points among children in the 2019 birth cohort was attributable to PM_2.5_ pollution. Across these children’s lifetimes, these losses of cognitive function will result in economic losses (reduced income) of $2.7 billion. Many of the PM_2.5_ exposures responsible for these adverse health effects occurred at exposure levels below the EPA standard of 9.0 μg/m³.

Our findings mirror those of a 2018 study of air pollution’s health impacts in Allegheny County, which found that up to 12% of total deaths in the county, or between 770 and 1640 deaths per year in the years 2012–2014, could be attributed to then-current levels of PM_2.5_ pollution [39]. As in our study, that report found that many of the pollution-related deaths in Allegheny County occurred at exposure levels below National Ambient Air Quality Standard (NAAQS) for PM_2.5_ pollution [34], thus documenting that current federal air pollution standards are set too high and do not adequately protect health.

Data from this study show that the highest rates of disease and death attributable to PM_2.5_ pollution in southwestern Pennsylvania occur in the region’s most highly polluted census tracts, especially in areas of Allegheny County to the south and east of Pittsburgh. These are areas where previous studies have documented high levels of pollution by black carbon and hazardous air pollutants [40, 41]. Lowest rates of all-cause and disease-specific mortality attributable to PM_2.5_ pollution and lowest regional levels of air pollution occurred in rural areas of Washington and Lawrence Counties.

These findings add to a growing literature demonstrating that air pollution is a major, insufficiently recognized cause of heart disease, pulmonary disease, and stroke—impacts that too often are ascribed solely to smoking, other personal behaviors, or “old age” without recognition that they can also be triggered by air pollution and therefore prevented by policies and enforcement actions that improve air quality [55, 56]. While the highest frequency of pollution-related cardiopulmonary disease and death among adults in the Pittsburgh MSA occurred in the most highly polluted census tracts, many of these health impacts occurred in census tracts where the annual mean PM_2.5_ concentration was below the EPA standard of 9.0 μg/m³, further demonstrating that current federal air pollution standards are set too high to adequately protect health. Any attempt to relax, roll back, or weaken enforcement of current air pollution standards will further magnify these harms [57].

Our findings on the impacts of PM_2.5_ pollution on the health of infants and children add to the growing literature that air pollution is an important cause of disease in infancy and childhood [12–14].

Our finding that air pollution in the Pittsburgh MSA results in widespread reduction in children’s IQ has negative implications for the health and well-being of individual children, because loss of cognitive function is highly correlated with poor academic performance, lower scores on standardized tests, and decreased high-school graduation rates [58, 59]. This finding also has negative implications for the economic well-being and technological growth of the southwestern Pennsylvania region [49]. A downward shift in the mean IQ of all children in a region by even as little as 2 points results in a significant decrease in the number of highly intelligent children (defined as IQ scores above 130 points) and a corresponding increase in the number with IQ scores below 70 [60]. Reduction in the number of highly intelligent children results in loss of regional capacity for future leadership. And increase in the number of children with IQ scores below 70 will increase regional need for special education services and increase the number of future adults who have limited capacity to live independently or to attain competitive employment.

Our finding that the census block groups in southwestern Pennsylvania with the highest levels of social disadvantage experienced highest annual mean PM_2.5_ concentrations [54] is consistent with a large body of literature, including prior studies from Pittsburgh, showing that pollution exposures are not evenly distributed across populations and that low-income, minority, and marginalized communities are often disproportionately heavily exposed, and are therefore at significantly increased risk of pollution-associated disease and death [61, 62]. Our findings here align with those of the above cited 2018 study of air pollution’s health impacts in Allegheny County, which found that townships with high percentages of minorities (>30% Black, Hispanic, Asian American, American Indian, or Alaska Native) had 18% higher rates of PM_2.5_-attributable deaths compared to those with lower percentages of minorities (<10%) [39]. These inequitable patterns of pollution exposure and disease are manifestations of environmental injustice and are the consequences of long-standing structural inequities such as real estate redlining and the disproportionate siting of polluting industrial facilities in predominantly minority and low-income communities [63].

Our analysis has several limitations. One of these is that we examined only PM_2.5_ pollution_,_ which is but one of the many components of air pollution. This limitation may be particularly important in a heavily industrialized region such as southwestern Pennsylvania, where point sources of industrial pollution, such as steel mills, coke works, and chemical plants, release multiple air pollutants in addition to PM_2.5_ including black carbon, sulfur oxides (SO_X_), nitrogen oxides (NO_X_), benzene, and other hazardous chemical pollutants. In defense of our reliance on PM_2.5_, it is the best studied component of air pollution and the component most reliably linked to adverse health effects.

A second limitation is that we did not consider the chemical composition of airborne particulate pollution in Pittsburgh but looked only at PM_2.5_ concentrations. Carbon-rich particulates from industrial point sources, such as coke ovens, may have different toxicity than particulates from sources such as automotive emissions.

Another limitation is that we relied solely on annual mean concentrations of PM_2.5_. While this approach is widely used in epidemiologic studies of air pollution and has proven valuable in identifying and quantifying many of PM_2.5_ pollution’s health effects [4–11], it can miss the acute health impacts of short-term pollution spikes [64]. Short-term (hours to days) increases in PM_2.5_ levels are associated with increased risk for hypertension, atrial fibrillation, myocardial infarction, stroke, and cardiovascular death. Risk of death increases by 0.1–4.0% with each 10 μg/m^3^ increase in PM_2.5_ concentration [65–67]. Past and recent experience in southwestern Pennsylvania documents the health impacts of short-term fluctuations in air pollution levels. The intense, short-term spike in air pollution that occurred during the Donora smog of 1948 resulted in multiple deaths and hospitalizations [36]. More recently, the large fire and subsequent failure of pollution control at the Clairton Coke Works in 2018 resulted in exacerbations of acute asthma in nearby communities [68].

On the positive side, short-term improvements in air quality in southwestern Pennsylvania have been shown to benefit both adult and child health. This was seen in the sharp reduction in emergency room visits for cardiac symptoms among adults and the 41% decrease in emergency room visits for pediatric asthma that followed the 2016 closing of the Shenango Coke Works [69, 70].

Another limitation in our analysis is that we utilized air pollution estimates from 2016 and data on disease and death for 2019. More recent and concomitant data would have been optimal, but these were the most recent data available to us.

Another potential limitation is that we had only one residential address per person. We were thus unable to account for residential relocation, which may have led to exposure misclassification.

The striking elevations in PM_2.5_ concentrations we observed in certain census tracts in Allegheny County, where annual mean levels were as high as 15.90 μg/m^3^, show that air pollution enforcement in Allegheny County is not adequate. Such elevations are clear violations of the federal Clean Air Act. Poor control of air pollution has been shown in studies conducted elsewhere to correlate with political corruption [71].

Disease and premature death caused by air pollution can be prevented. The 75% reduction in air pollutant emissions achieved in the United States since passage of the Clean Air Act in 1970 [42] and similar reductions seen in other countries demonstrate clearly that air pollution can be controlled by laws, regulations, and technologies that are based on science, backed by enforcement, and encouraged by incentives [1]. These improvements in air quality have been shown in multiple studies to improve health and well-being, prevent disease, extend longevity, and save lives [9, 18, 69, 70, 72].

Interventions to improve air quality are highly cost-effective. Each dollar invested in air pollution control in the United States since 1970 is estimated to have yielded an economic benefit of $30 through reducing health-care costs and increasing the economic productivity of a healthier, longer-lived population [42]. Attempts to provide industry with short-term economic benefit by rolling back and not enforcing air pollution standards [57] will impose substantial economic losses on citizens and governments.

Enduring control of air pollution will be most effectively achieved by wide-scale transition away from fossil fuels to clean, renewable energy (Table 6). Two very encouraging recent developments increase the likelihood of such a transition. The first is an almost 500% increase since 2010 in the fraction of US electricity generated from wind and solar power, with the result that in 2021, for the first time, investment in renewables surpassed all spending on oil and gas exploration [73]. The levelized cost of offshore wind power has fallen by 63%, the cost of solar power by 90%, and the cost of batteries by over 90% [74]. It is now cheaper in many parts of the United States to produce electricity from renewables than from any fossil fuel [75].

**Table 6:** Recommendations to improve air quality, prevent pollution-related disease, and save lives.

**Community-Level Recommendations:** Convert all municipal vehicle fleets—cars, trucks, buses—to hybrid and fully electric vehicles. Place solar panels on the roofs of municipal buildings. Preferentially purchase electricity produced by renewable energy. Block construction of gas pipelines, compressor stations, and other components of the natural gas network. Prohibit gas hook-ups in new construction. Revise building codes to increase energy efficiency. **County-Level Recommendations:** Strictly enforce National Ambient Air Quality Standards for all criteria air pollutants and all hazardous air pollutants. Strictly enforce emission standards from all stationary and mobile sources. Add more air monitoring stations and increase the density of the ambient air monitoring network. There is particular need to prioritize placement of air monitoring stations in economically disadvantaged and socially vulnerable communities. Publish an annually updated, open-source, web-based air pollution emissions inventory in an easily accessible, interactive dashboard-style format. Create an open-access, web-based dashboard that annually tracks and publicizes information on pollution-related disease and death in each county, city, and town. **Federal-level recommendations** Tighten federal air quality standards for PM_2.5_ pollution to better protect health. The occurrence of disease, premature death, and cognitive impairment at PM_2.5_ pollution levels below current federal standards is clear evidence that these standards are not adequately protective of health. Current federal air pollution standards fail especially to protect children’s health. A critical next step will be to lower the National Ambient Air Quality Standard for PM_2.5_ pollution to at least 5 μg/M^3^, the level recommended by the World Health Organization. Reduce pollutant emissions by accelerating progress away from fossil fuels toward net zero carbon through a rapid, wide-scale, government-supported transition from away all fossil fuels—coal, gas, and oil—to clean, renewable energy. Two powerful tools for accelerating this transition are phase-outs of all governmental subsidies and tax breaks for the fossil fuel industry and increased incentives for wind and solar power. Expend and strengthen the national electric power grid to meet rising national energy needs and to accommodate the increasing adoption of wind and solar energy. Resist the temptation to increase reliance on nuclear power.

While current actions at the federal level in the United States are slowing the transition to clean energy [76], progress continues to made at the state and local level, and the longer-term trend away from fossil fuels and toward renewables appears inevitable, given continuing reductions in the costs of clean energy [75], accumulating data on the adverse health consequences of fossil-fuel-related air pollution[1], and escalating concern about the health and economic consequences of climate change [3].

The impediments to air pollution control in the United States and in southwestern Pennsylvania are no longer technical but rather are economic and political. Key to control of air pollution and prevention of its harms to health will be courageous and visionary political leaders, who heed the science, recognize pollution’s great dangers, and enforce the law.

## Conclusion

By linking individual vital records with satellite-derived air pollution estimates at the census-tract level, we find that an estimated 11.1%–12.5% of adult deaths in the Pittsburgh metropolitan area in 2019, adverse birth outcomes in over 400 children, widespread reductions in children’s IQ, and economic losses totaling $2.7 billion could be attributed to PM_2.5_ air pollution. Many of these health impacts occurred at PM_2.5_ exposure levels below the current EPA PM_2.5_ standard of 9.0 μg/m³. Public policies and strict enforcement that improve air quality in southwestern Pennsylvania will improve health, save lives, and enhance the region’s economic productivity.

## Additional Files

The additional files for this article can be found as follows:
