# Supplementary material for: Particulate Air Pollution, Disease, and Death in the Cities and Towns of Southwestern Pennsylvania
Source: Ann Glob Health. 2026 Jan 28;92(1):10. doi: 10.5334/aogh.5145 (PMC12862455; doi:10.5334/aogh.5145)
Supplement: Supplementary material. — Supplementary Tables. [file agh-92-1-5145-s1.pdf]

AOGH 5145. Particulate Air Pollution, Disease and Death in the Cities and Towns of Southwestern Pennsylvania. Whitman *et al.*

SUPPLEMENTARY TABLES

Supplementary Table 1. Health Outcomes Included in the Analysis

**Adult Mortality**	**ICD-10 Code**
All-Cause	A00 – Z99
Lung Cancer	C34
Ischemic Heart Disease	I20 – I25
Myocardial Infarction	I21 – I22
Chronic Obstructive Pulmonary Disease	J40 – J44
**Adverse Birth Outcomes**	**Definition**
Preterm Birth	<37 weeks' gestation
Low Birth Weight	< 2500 grams
**Pediatric Outcomes**	
IQ loss	

Supplementary Table 2. $PM_{2.5}$ Mortality Outcome Correlation Coefficients Used in the Analysis

Mortality Type	Author	Pollutant	Study Year	Gender	Start Age	End Age	Beta	Standard Deviation	Reference
All Cause	Laden	PM2.5	2006	All	25	99	0.015	0.042	(9)
All Cause	Lepeule	PM2.5	2012	All	25	99	0.013	0.031	(46)
Lung Cancer	Laden	PM2.5	2009	All	25	74	0.024	0.144	(9)
Lung Cancer	Gharibvand	PM2.5	2017	All	30	99	0.036	0.129	(47)
Ischemic Heart Disease	Krewski	PM2.5	2009	All	30	99	0.022	0.002	(48)
Myocardial Infarction	Alexeeff	PM2.5	2021	All	18	99	0.008	0.040	(11)
COPD	Krewski	PM2.5	2009	All	30	99	0.012	0.003	(48)

Supplementary Table 3. $PM_{2.5}$ and Birth Outcomes Correlation Coefficients Used in the Analysis

Endpoint	Author	Pollutant	Study Year	Gender	Start Age	End Age	Beta	Standard Deviation	Reference
Low birth weight	Ghosh	$PM_{2.5}$	2021	All	0	0	0.010	0.002	(13)
Preterm birth	Ghosh	$PM_{2.5}$	2021	All	0	0	0.011	0.003	(13)
Still birth	Zhang	$PM_{2.5}$	2021	All	0	0	0.009	0.004	(14)

Supplementary Table 4. Estimated Annual Deaths Preventable if PM2.5 Levels are Reduced to 5 μg/m^3

	**All-Cause Mortality (A00 – Z99)**				**Lung Cancer (C34)**		**Ischemic Heart Disease (IHD) (I20 – I25)**		**Myocardial Infarction (subset of IHD) (I21)**	
	Lepeule (2012)		Laden (2006)		Gharibvand (2017)		Krewski (2009)		Alexeeff (2021)	
**County**	**Counts**	**Percent Attributable**	**Counts**	**Percent Attributable**	**Counts**	**Percent Attributable**	**Counts**	**Percent Attributable**	**Counts**	**Percent Attributable**
Allegheny	771	5.89%	870	6.65%	100	15.36%	194	9.50%	16	3.52%
Armstrong	36	4.48%	41	5.06%	6	11.97%	7	7.31%	1	2.77%
Beaver	91	4.34%	103	4.91%	12	11.46%	19	7.04%	2	2.58%
Butler	88	4.35%	99	4.91%	12	11.35%	18	7.09%	2	2.57%
Fayette	64	3.78%	72	4.27%	10	9.93%	16	6.19%	1	2.27%
Lawrence	46	4.19%	52	4.73%	6	10.98%	9	6.71%	1	2.46%
Washington	100	4.01%	113	4.52%	17	10.35%	22	6.85%	2	2.39%
Westmoreland	220	4.97%	249	5.61%	29	12.86%	48	8.00%	5	2.87%
**Pittsburgh MSA**	**1416**	5.11%	**1599**	5.77%	**191**	13.18%	**332**	8.38%	**30**	3.02%
